# Supplementary material for: Severe Acute Kidney Injury in Critically Ill Patients with COVID-19 Admitted to ICU: Incidence, Risk Factors, and Outcomes
Source: J Clin Med. 2021 Mar 15;10(6):1217. doi: 10.3390/jcm10061217 (PMC7998509; doi:10.3390/jcm10061217)
Supplement: Supplementary file 1 [file jcm-10-01217-s001.pdf]

Severe Acute Kidney Injury in Critically Ill patients with COVID-19 admitted to ICU: Incidence, Risk Factors, and Outcomes

Table S1. Factors associated with severe AKI (stages 2 and 3) in multivariable logistic regression analysis

Variables	Odds ratio	95% Confidence interval	*P*-value
Age, year	1.09	1.01-1.17	**0.022**
SOFA score	0.96	0.75-1.24	0.78
Chronic kidney disease, (reference: no chronic kidney disease)	-	-	-
Diabetes mellitus, (reference: no diabetes mellitus)	2.47	0.63-9.62	0.19
Essential hypertension, (reference: no essential hypertension)	0.81	0.17-3.79	0.79
Lowest mean arterial pressure, mmHg	0.99	0.93-1.06	0.80
D-dimer, $\mu g.mL^{-1}$	1.02	0.65-1.62	0.92
Invasive mechanical ventilation	16.00	1.50-170.03	**0.022**
Creatinine at ICU admission, $\mu mol.L^{-1}$	1.03	1.006-1.063	**0.018**
Vasopressor support, (reference: no vasopressor support)	1.04	0.15-7.01	0.97
Leucocytes count, $\times 10^9.l^{-1}$	1.02	0.91-1.13	0.76
Extracorporeal membrane oxygenation, (refer: no)	14.75	2.06-105.52	**0.007**

SOFA, Sequential Organ Failure Assessment; ICU, intensive care unit.

$p \leq 0.05$ was considered statistically significant.

goodness-of-fit test: p= 0.87

Akaike Information Criteria (AIC): 86.47992

Bayesian Information Criteria (BIC): 117.6214

Table S2. Factors associated with AKI stages 2 and 3 (multivariable logistic regression analysis)

Variables	Odds ratio	95% Confidence interval	*P*-value
Age, year	1.09	1.01-1.17	**0.022**
SOFA score	0.96	0.75-1.24	0.78
Chronic kidney disease, (reference: no)	-	-	-
Diabetes mellitus, (reference: no)	2.47	0.63-9.62	0.19
Essential hypertension	0.81	0.17-3.79	0.79
Lowest mean arterial pressure, mmHg	0.99	0.93-1.06	0.80
D-dimer, $\mu g.mL^{-1}$	1.02	0.65-1.62	0.92
Invasive mechanical ventilation	16.00	1.50-170.03	**0.022**
Creatinine, $\mu mol.L^{-1}$	1.03	1.006-1.063	**0.018**
Vasopressor support, (reference: no)	1.04	0.15-7.01	0.97

Leucocytes count, × $10^9.l^{-1}$	1.02	0.91-1.13	0.76
Extracorporeal membrane oxygenation, (refer: no)	14.75	2.06-105.52	**0.007**

SOFA, Sequential Organ Failure Assessment.

$p \leq 0.05$ was considered statistically significant.

goodness-of-fit test: p= 0.87

Akaike Information Criteria (AIC): 86.47992

Bayesian Information Criteria (BIC): 117.6214

Table S3. Factors associated with hospital mortality (multivariable logistic regression analysis)

Variables	Odds ratio	95% Confidence interval	*P*-value
Severe AKI (stages 2 and 3), (reference: no AKI/AKI stage 1)	10.17	3.06- 33.76	**< 0.001**
Age, year	1.00	0.96-1.04	0.83
SAPS II	1.03	0.99-1.06	0.10
Comorbidities, (reference: no comorbidities)	1.45	0.45-4.63	0.53
Platelet count, × $10^9.l^{-1}$	0.992	0.987-0.999	**0.02**

SAPS, Simplified Acute Physiology Score; AKI, acute kidney injury.

$p \leq 0.05$ was considered statistically significant.

goodness-of-fit test: p= 0.74

Table S4. Factors associated with hospital mortality (multivariable logistic regression analysis)

Variables	Odds ratio	95% Confidence interval	*P*-value
Severe AKI (stages 2 and 3), (reference: no AKI/AKI stage 1)	9.25	2.56- 33.41	**0.001**
Age, year	1.01	0.97-1.06	0.59
SAPS II	1.01	0.98-1.04	0.39
Comorbidities, (reference: no comorbidities)	1.46	0.50-4.22	0.49
Invasive mechanical ventilation	1.03	0.26- 4.09	0.96

SAPS, Simplified Acute Physiology Score; AKI, acute kidney injury.

$p \leq 0.05$ was considered statistically significant.

goodness-of-fit test: p= 0.52

Table S5. Factors associated with hospital mortality (multivariable logistic regression analysis)

Variables	Odds ratio	95% Confidence interval	*P*-value
Severe AKI (stages 2 and 3), (reference: no AKI/AKI stage 1)	13.46	3.25- 55.68	**< 0.001**
Age, year	1.00	0.97-1.05	0.72
SAPS II	0.99	0.96-1.03	0.83
Comorbidities, (reference: no comorbidities)	1.71	0.55-5.30	0.35
Ferritin, $\mu g.L^{-1}$	1.00	1.00-1.00	0.13

SAPS, Simplified Acute Physiology Score; AKI, acute kidney injury.

$p \leq 0.05$ was considered statistically significant.

goodness-of-fit test: p= 0.33

Table S6. Factors associated with hospital mortality (multivariable logistic regression analysis)

Variables	Odds ratio	95% Confidence interval	*P*-value
Severe AKI (stages 2 and 3), (reference: no AKI/AKI stage 1)	20.65	3.87- 110.11	**< 0.001**
Age, year	0.99	0.95-1.04	0.80
SAPS II	1.00	0.97-1.04	0.85
Comorbidities, (reference: no comorbidities)	1.38	0.43-4.44	0.59
Interleukin 6, $ng.L^{-1}$	1.00	1.00-1.00	0.12

SAPS, Simplified Acute Physiology Score; AKI, acute kidney injury.

$p \leq 0.05$ was considered statistically significant.

goodness-of-fit test: p= 0.67

Table S7. Factors associated with hospital mortality (multivariable logistic regression analysis)

Variables	Odds ratio	95% Confidence interval	*P*-value
Severe AKI (stages 2 and 3), (reference: no AKI/AKI stage 1)	8.68	2.33- 32.30	**0.001**
Age, year	1.01	0.96-1.07	0.59
SAPS II	1.01	0.98-1.04	0.49
Comorbidities, (reference: no comorbidities)	1.24	0.39-3.97	0.71
PaO_2/FiO_2, mmHg	0.99	0.98-1.00	0.09

SAPS, Simplified Acute Physiology Score; AKI, acute kidney injury; PaO_2, arterial oxygen pressure.

$p \leq 0.05$ was considered statistically significant.

goodness-of-fit test: p= 0.70

Table S8. Factors associated with hospital mortality (multivariable logistic regression analysis)

Variables	Odds ratio	95% Confidence interval	*P*-value
Severe AKI (stages 2 and 3), (reference: no AKI/AKI stage 1)	9.09	2.64- 31.31	**< 0.001**
Age, year	1.01	0.97-1.06	0.47
SAPS II	1.00	0.96-1.03	0.84
Comorbidities, (reference: no comorbidities)	1.41	0.45-4.39	0.55
Lactate, $mmol.L^{-1}$	2.23	0.99-4.99	0.051

SAPS, Simplified Acute Physiology Score; AKI, acute kidney injury.

$p \leq 0.05$ was considered statistically significant.

goodness-of-fit test: p= 0.78

Table S9. Factors associated with hospital mortality (multivariable logistic regression analysis)

Variables	Odds ratio	95% Confidence interval	*P*-value
Severe AKI (stages 2 and 3), (reference: no AKI/AKI stage 1)	6.62	1.85- 23.70	**0.004**
Age, year	1.00	0.95-1.06	0.83
SAPS II	1.00	0.97-1.03	0.74
Comorbidities, (reference: no comorbidities)	1.77	0.62-5.05	0.29
Vasopressor support, (reference: no vasopressors)	3.52	0.71- 17.38	0.12

SAPS, Simplified Acute Physiology Score; AKI, acute kidney injury.

$p \leq 0.05$ was considered statistically significant.

goodness-of-fit test: p= 0.46

Table S10. Factors associated with hospital mortality (multivariable logistic regression analysis)

Variables	Odds ratio	95% Confidence interval	*P*-value
Severe AKI (stages 2 and 3), (reference: no AKI/AKI stage 1)	8.87	2.72- 28.91	**< 0.001**
Age, year	1.02	0.97-1.06	0.39
SAPS II	1.00	0.97-1.03	0.90
Comorbidities, (reference: no comorbidities)	1.69	0.57-5.02	0.34
Leucocytes count, $\times 10^{9}.l^{-1}$	1.10	0.98-1.23	0.12

SAPS, Simplified Acute Physiology Score; AKI, acute kidney injury.

$p \leq 0.05$ was considered statistically significant.

goodness-of-fit test: p= 0.36

Table S11. Factors associated with hospital mortality (multivariable logistic regression analysis)

Variables	Odds ratio	95% Confidence interval	*P*-value
Severe AKI (stages 2 and 3), (reference: no AKI/AKI stage 1)	7.40	2.03- 26.93	**0.002**
Age, year	1.02	0.97-1.06	0.42
SAPS II	1.02	0.99-1.04	0.23
Comorbidities, (reference: no comorbidities)	1.81	0.56-5.36	0.32
Extracorporeal membrane oxygenation, (refer: no)	4.98	0.65- 37.85	0.12

SAPS, Simplified Acute Physiology Score; AKI, acute kidney injury.

$p \leq 0.05$ was considered statistically significant.

goodness-of-fit test: p= 0.85

Table S12. Factors associated with hospital mortality (multivariable logistic regression analysis)

Variables	Odds ratio	95% Confidence interval	*P*-value
Severe AKI (stages 2 and 3), (reference: no AKI/AKI stage 1)	9.46	2.82- 31.76	**< 0.001**
Age, year	1.02	0.97-1.06	0.36
SAPS II	1.01	0.98-1.04	0.46
Comorbidities, (reference: no comorbidities)	1.29	0.42-4.00	0.66
Hydroxychloroquine, (reference: no hydroxychloroquine)	0.39	0.12-1.28	0.12

SAPS, Simplified Acute Physiology Score; AKI, acute kidney failure.

$p \leq 0.05$ was considered statistically significant.

goodness-of-fit test: p= 0.18

Table S13. Factors associated with hospital length of stay in multivariable competing-risks regression analysis

Variables	Sub-Hazards ratio	95% Confidence interval	*P*-value
Severe AKI (stages 2 and 3), (reference: no AKI/AKI stage 1)	0.26	0.14- 0.51	**< 0.001**
Age, year	1.00	0.97-1.02	0.91
SOFA score	1.06	0.98-1.15	0.16
Comorbidities, (reference: no comorbidities)	0.94	0.52-1.68	0.98
Platelet count, × $10^9.l^{-1}$	1.00	1.00-1.00	0.86
Invasive mechanical ventilation, (reference: no invasive mechanical ventilation)	0.62	0.26- 1.50	0.28
Ferritin, $\mu g.L^{-1}$	1.00	1.00-1.00	0.20
Interleukin 6, $ng.L^{-1}$	1.00	1.00-1.00	0.20
PaO_2/FiO_2, mmHg	1.00	0.99-1.00	0.61
Lactate, $mmol.L^{-1}$	0.61	0.39-0.94	**0.026**
Vasopressor support, (reference: no vasopressors)	0.31	0.12- 0.79	**0.014**
Leucocytes count, × $10^9.l^{-1}$	0.97	0.91-1.04	0.39
Extracorporeal membrane oxygenation, (refer: no extracorporeal membrane oxygenation)	0.18	0.06- 0.51	**0.001**

SOFA, Sequential Organ Failure Assessment; AKI, acute kidney injury; PaO_2, arterial oxygen pressure; FiO_2, inspiratory oxygen fraction.

$p \leq 0.05$ was considered statistically significant.
